# Correction: ‘Multidimensional plasticity in the Glanville fritillary butterfly: larval performance is temperature, host and family specific’ (2020), by Nadja *et al.*

**DOI:** 10.1098/rspb.2023.2282

**Published:** 2023-11-29

**Authors:** Verspagen Nadja, Ikonen Suvi, Saastamoinen Marjo, van Bergen Erik

*Proc. R. Soc. B* **287**: 20202577 (Published online 16 December 2020). (https://doi.org/10.1098/rspb.2020.2577)

While conducting additional research on a similar topic, we discovered an error in the computation of relative fat content in the original article. In short, in the methods section, we describe the correct formula to calculate this: relative fat content = (dry weight – fat-free dry weight)/fat-free dry weight. However, we erroneously used fresh weight instead of dry weight to compute the relative fat content for our analyses, resulting in lower fat content values. We have repeated the analyses with the correct values for relative fat content and the mistake does not affect the results or conclusions of the article. Here, we present a new version of Figure 2 and Table S5. This has now been corrected.

**Figure 2.**
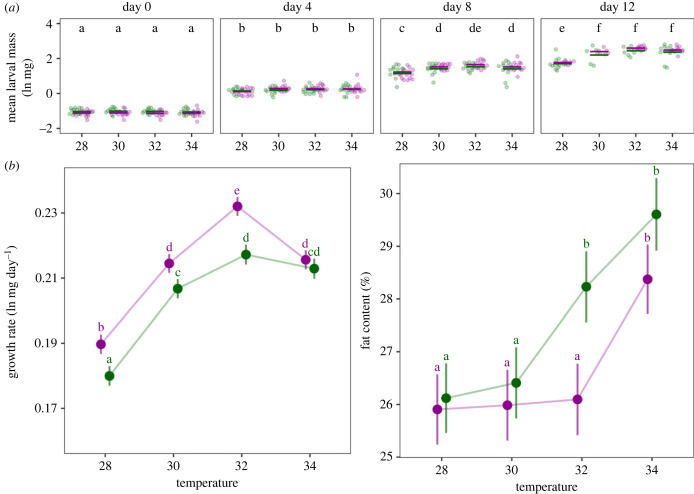
Environmentally induced variation in life-history traits. (*a*) Dots depict the mean larval mass, logtransformed and corrected for number of individuals, in each thermal environment (x-axis), for Plantago (green) and Veronica (purple), on four assessment days (from left to right: day 0 [i.e. 2nd instar mass], day 4, day 8 and day 12). Significant differences between thermal treatments (Tukey's HSD, α = 0.05) are indicated by different letters. Details of the statistical test can be found in electronic supplementary material, table S3. (*b*) Model-estimated marginal means for the individual growth rates (left; R2 = 0.5964) and the relative fat content (right; R2 = 0.4992). Error bars represent 95% confidence intervals and significant differences between groups (Tukey's HSD, α = 0.05), averaged over the families, are indicated by different letters. Details of statistical tests can be found in electronic supplementary material, tables S3 and S4.

**Reference**

1. Verspagen N, Ikonen S, Saastamoinen M, van Bergen E. 2020 Multidimensional plasticity in the Glanville fritillary butterfly: larval performance is temperature, host and family specific. *Proc. R. Soc. B* **287**, 20202577 Issue: 1941. (doi:10.1098/rspb.2020.2577)

