# Supplementary material for: Multidimensional plasticity in the Glanville fritillary butterfly: larval performance is temperature, host and family specific
Source: Proc Biol Sci. 2020 Dec 16;287(1941):20202577. doi: 10.1098/rspb.2020.2577 (PMC7779508; doi:10.1098/rspb.2020.2577)
Supplement: Supplementary Materials [file rspb20202577supp1.pdf]

SUPPLEMENTARY MATERIALS

TITLE:

Multidimensional plasticity in the Glanville fritillary butterfly: larval performance is temperature, host and family specific.

AUTHORS:

Nadja Verspagen, Suvi Ikonen, Marjo Saastamoinen and Erik van Bergen.

JOURNAL:

Proceedings of the Royal Society B

DOI:

10.1098/rspb.2020.2577

CONTENTS

Figure S1: Experimental design.

Figure S2: Mean development time and diapause mass per developmental temperature and host plant.

Figure S3: Family specific responses to host plant.

Figure S4: Variance in larval growth rates within families.

Figure S5: Minimum, mean and maximum temperatures in Åland.

Table S1: Background of larvae used in the experiment.

Table S2: Generalised linear mixed effects model for endpoint survival.

Table S3: Linear model for mean clutch mass.

Table S4: Linear model for individual growth rates.

Table S5: Linear model for individual fat content.

Table S6: Linear model for individual development time.

Table S7: Linear model for individual diapause mass.

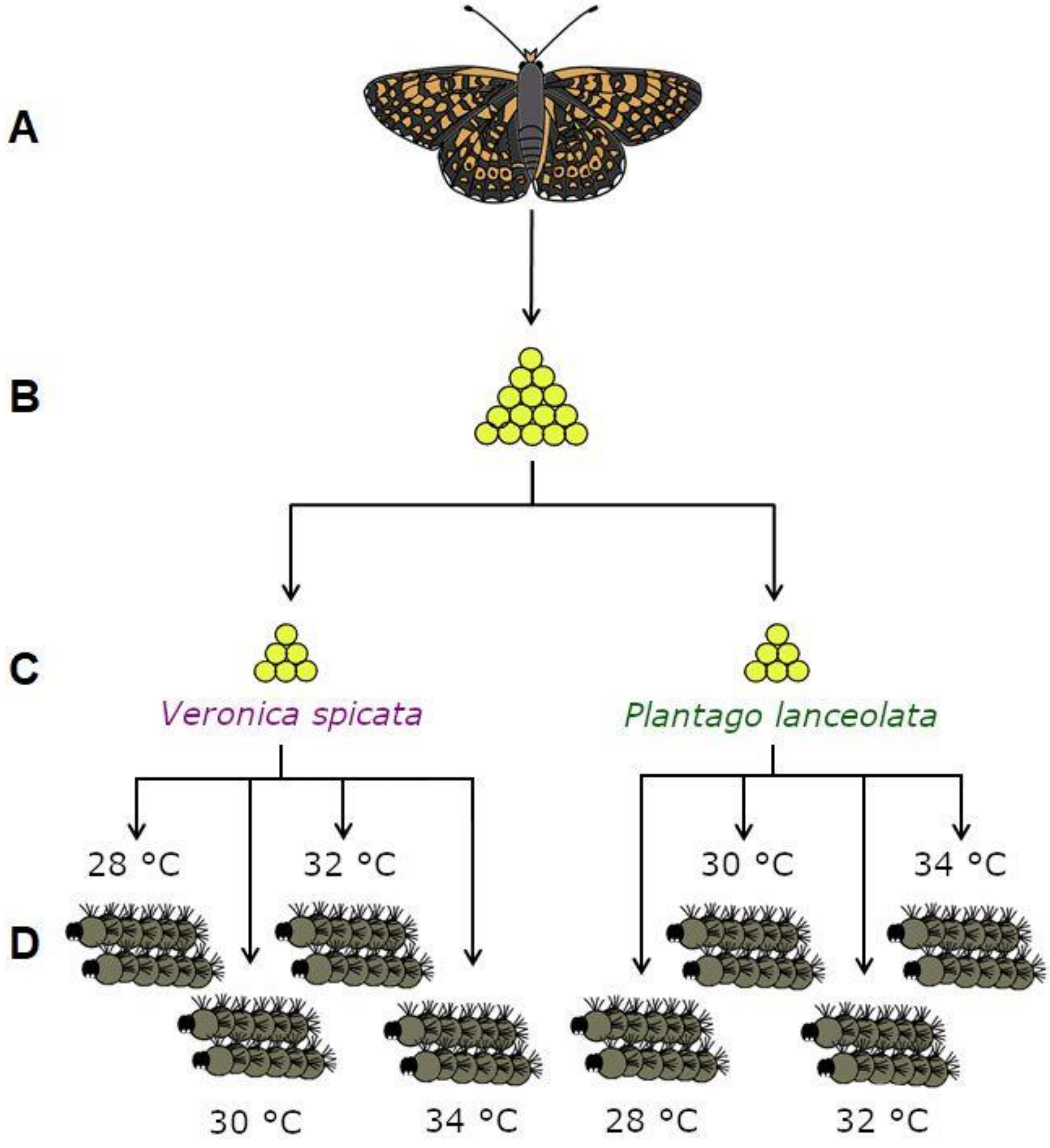

Figure S1: Schematic outline of the experimental design. The egg clutch of one female (**A** + **B**) was split over two host plants (**C**), and further divided over four temperature treatments upon transitioning to the second instar (15 larvae per treatment, **D**). This was done for offspring of 15 females from different families.

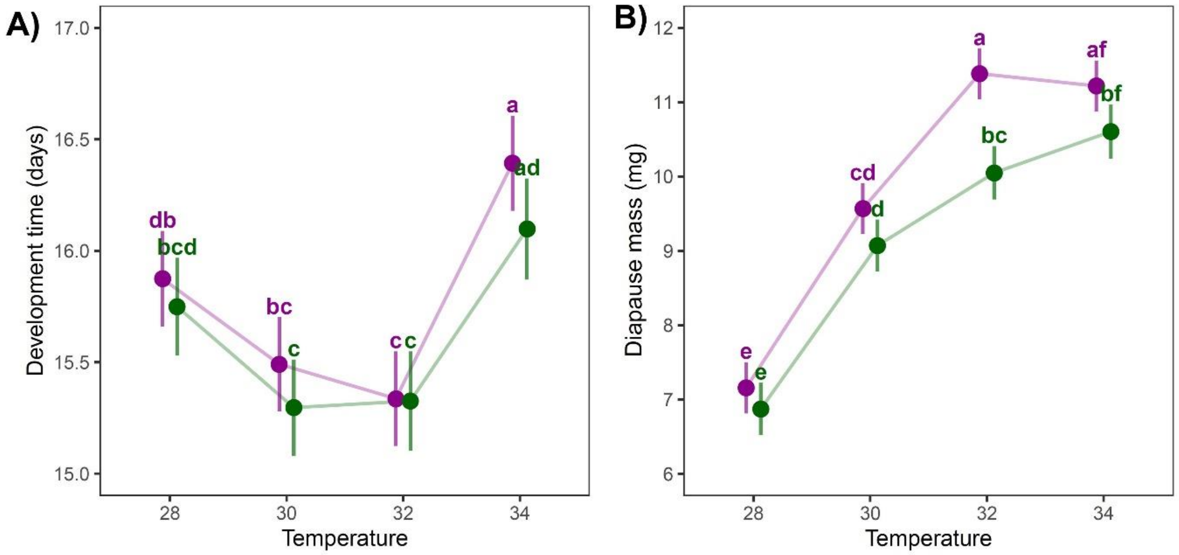

Figure S2: Environmentally induced variation in life history traits. Model-estimated marginal means for the individual **A**) development time (R^2 = 0.3941) and **B**) diapause mass (R^2 = 0.5283). Error bars represent 95% confidence intervals and significant differences between groups (Tukey's HSD, α = 0.05), averaged over the families, are indicated by different letters. Details of statistical tests can be found in Tables S6 and S7.

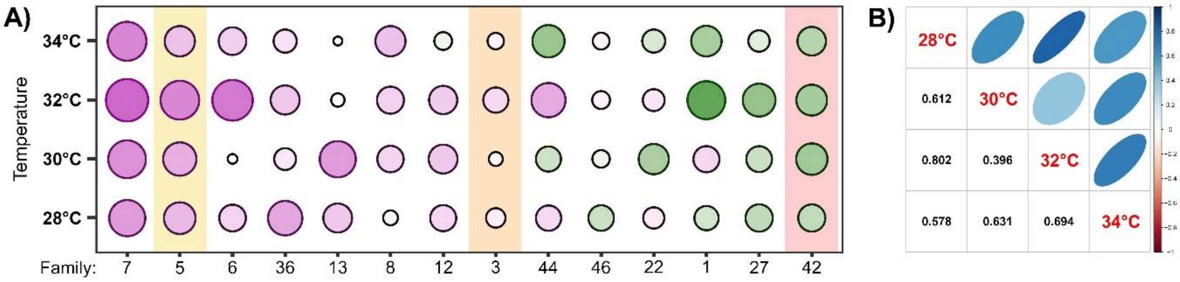

Figure S3: Family-specific responses to the host plant. **A**) Some families consistently achieve higher growth rates on *Veronica* while other families, regardless of the thermal environment, grow consistently faster on *Plantago.* The size of the symbol depicts the magnitude of the host-induced response, with steeper reaction norm slopes represented by larger symbols. The direction of the response to the host plant is represented by the colour of the symbol, with higher growth rates on *Veronica* depicted in purple and higher growth rates on *Plantago* in green. Highlighted families correspond to those given in figure 3A of the main text. **B**) Pearson's correlation coefficients among the host-induced reaction norm slopes were positive and ranged between 0.4 and 0.8. The host-induced responses are therefore family-specific and largely consistent across thermal environments.

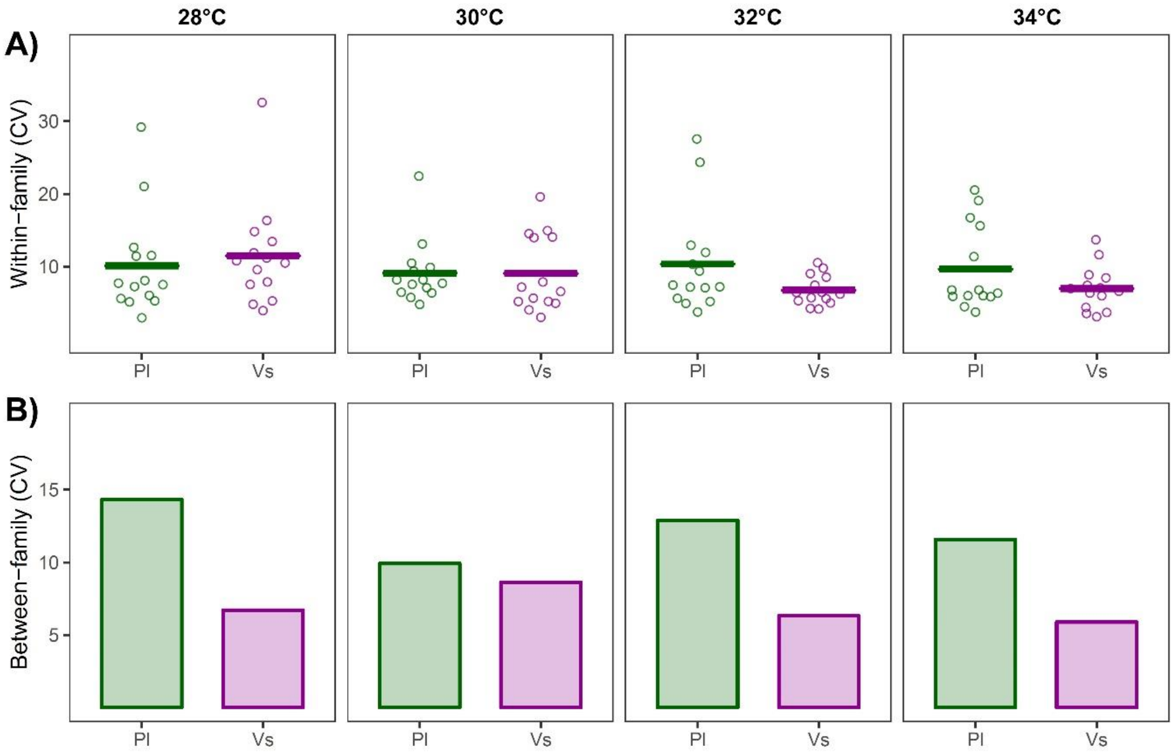

Figure S4: A) Variance in larval growth rates within families (CV; standard deviation divided by the mean for each family) was similar between host treatments. **B**) Utilising *Plantago* as a host plant resulted in higher variance in larval growth rates across families (CV; standard deviation of family means divided by the global mean growth rate, calculated for each temperature treatment separately)

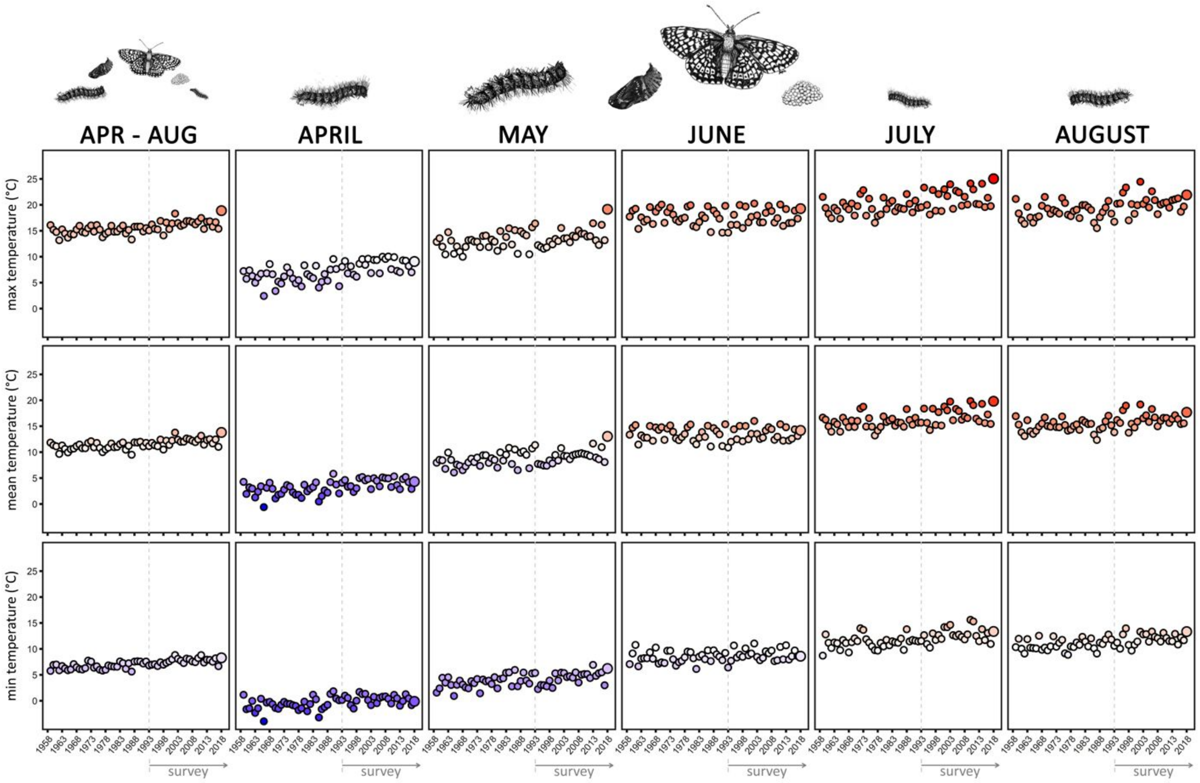

Figure S5: Minimum, mean and maximum temperatures in Åland in the months April until August for years between 1958 and 2018. Symbol colour gradient indicates temperature, with cold temperatures represented by blue and warm temperatures indicated by red. The pictures above the panels show the presence of butterfly life-stages per time period, with post diapause larvae being present in April and May, after which they pupate and emerge as adults who lay their eggs in June. Pre-diapause larvae then develop in July and August. Temperature data was derived from the Jomala climate station database in Åland. Illustrations courtesy of Luisa Woestmann.

Table S1: Background of larvae used in the experiment including family of the mother and the clutch number (1 = first clutch of the female, 2 = second clutch of the female). Abbreviation Vs stands for host plant *Veronica spicata*, Pl for host plant *Plantago lanceolata.*

Mother	Clutch number							
	28 °C		30 °C		32 °C		34 °C	
	Vs	Pl	Vs	Pl	Vs	Pl	Vs	Pl
1	1	1	1	2	1	1	1	1
3	1	1	1	1	1	1	1	1
5	1	1	1	1	1	1	1	1
6	1	1	1	1	2	1	1	1
7	1	1	1	1	1	1	1	1
8	1	1	1	1	1	1	1	1
12	1	1	1	1	1	1	2	1
13	1	1	1	1	1	1	1	1
22	1	1	1	1	1	1	1	1
27	1	1	1	1	1	2	1	1
36	1	1	1	1	1	1	1	1
42	1	1	1	1	1	1	1	1
44	1	1	1	1	1	1	1	1
46	1	1	1	1	1	1	1	1

Table S2: Generalised linear mixed effects model (GLMM) for endpoint survival data. **A**) Anova table for the model. **B**) Model-estimated marginal means, as well as upper and lower confidence limits, for all experimental treatments. Significant differences between host plants within each thermal environment (Tukey's HSD, α = 0.05) were obtained using the package *emmeans*.

**A**	Df	ChiSq	P value
Temperature	3	3.6068	0.3072
Plant	1	18.0706	< 0.0001
Temperature:Plant	3	1.0116	0.7985

**B** Temperature	Plant	Survival probability	LCL	UCL	Tukey’s HSD
28 °C	Pl	0.933	0.876	0.965	0.1679
28 °C	Vs	0.960	0.917	0.981	
30 °C	Pl	0.941	0.888	0.970	0.0528
30 °C	Vs	0.975	0.941	0.990	
32 °C	Pl	0.909	0.840	0.950	0.0066
32 °C	Vs	0.968	0.929	0.985	
34 °C	Pl	0.905	0.834	0.947	0.0136
34 °C	Vs	0.960	0.917	0.981	

Pl = *Plantago*; Vs = *Veronica*

Table S3: Linear model for mean clutch mass (related to Figure 2A in the main text). **A**) Minimum adequate model (in bold) was obtained using the *step()* function, starting from the full model. **B**) Anova table for the minimum adequate model. **C**) Model-estimated marginal means, as well as upper and lower confidence limits, for all experimental treatments. Significant differences between experimental groups (Tukey's HSD, α = 0.05), averaged over the families and host plants, were obtained using the package *emmeans* and are indicated by different letters.

**A**		AIC
M_{full}	Ln(mass) ~ t * T * HP	-1162.5
M_1	Ln(mass) ~ t + T + HP + t:T + t:HP + T:HP	-1177.6
**M_{final}**	**Ln(mass) ~ t + T + HP + t:T + t: HP**	**-1179.8**
M_3	Ln(mass) ~ t + T + HP + t:T + T:HP	-1171.2
M_4	Ln(mass) ~ t + T + HP + t:HP + T:HP	-1107.7
M_5	Ln(mass) ~ t + T + HP + t:T	-1173.6
M_6	Ln(mass) ~ t + T + HP + t: HP	-1111.8

t = time-point; T = temperature; HP = host plant.

**B**	Df	Sum Sq	Mean Sq	F value	P value	% exp
Time-point	3	615.2663	205.0888	3768.1423	< 0.0001	94.9792
Temperature	3	5.2870	1.7623	32.3799	< 0.0001	0.8162
Plant	1	0.1098	0.1098	2.0169	0.1564	0.0169
Time-point:Temperature	9	5.1522	0.5725	10.5182	< 0.0001	0.7954
Time-point:Plant	3	0.6399	0.2133	3.9190	0.0089	0.0988
Residuals	392	21.3354	0.0544			3.2936

**C** Temperature	Time-point	Mean	LCL	UCL	Group
28 °C	0	-1.0789	-1.16619	-0.99152	a
30 °C	0	-1.0636	-1.15095	-0.97628	a
32 °C	0	-1.0795	-1.16684	-0.99217	a
34 °C	0	-1.0926	-1.17996	-1.00529	a
28 °C	4	0.1221	0.03477	0.20944	a
30 °C	4	0.2316	0.14423	0.31890	a
32 °C	4	0.2416	0.15425	0.32891	a
34 °C	4	0.2509	0.16352	0.33819	a
28 °C	8	1.1837	1.09635	1.27102	b
30 °C	8	1.4689	1.38152	1.55619	bc
32 °C	8	1.5895	1.50220	1.67687	cg
34 °C	8	1.4655	1.37820	1.55287	cd
28 °C	12	1.7373	1.63179	1.84272	g

30 °C	12	2.2997	2.16625	2.43306	e
32 °C	12	2.5193	2.41542	2.62313	fe
34 °C	12	2.4235	2.32922	2.51788	e

Table S4: Linear model for individual growth rates (related to Figure 2B in the main text). **A**) Minimum adequate model (in bold) was obtained using the *step()* function, starting from the full model. **B**) Anova table for the minimum adequate model. **C**) Model-estimated marginal means, as well as upper and lower confidence limits, for all experimental treatments. Significant differences between experimental groups (Tukey's HSD, α = 0.05), averaged over the families, were obtained using the package *emmeans* and are indicated by different letters.

**A**		AIC
**M_{full}**	**Growth rate ~ F * T * HP**	**-11924.0**
M_2	Growth rate ~ F + T + HP + F:T + F:HP + T:HP	-11812.0

F = family; T = temperature; HP = host plant.

**B**	Df	Sum Sq	Mean Sq	F value	P value	% exp
Family	13	0.2363	0.0182	41.6611	< 0.0001	15.1482
Temperature	3	0.3266	0.1089	249.5829	< 0.0001	20.9423
Plant	1	0.0279	0.0279	63.9802	< 0.0001	1.7895
Family:Temperature	39	0.0664	0.0017	3.9012	< 0.0001	4.2555
Family:Plant	13	0.1829	0.0141	32.2507	< 0.0001	11.7265
Temperature:Plant	3	0.0086	0.0029	6.5948	0.0002	0.5534
Family:Temperature:Plant	39	0.0815	0.0021	4.7893	< 0.0001	5.2243
Residuals	1443	0.6295	0.0004			40.3603

**C** Temperature	Plant	Mean	LCL	UCL	Group
28 °C	Pl	0.1800	0.1770	0.1830	e
30 °C	Pl	0.2067	0.2038	0.2097	c
32 °C	Pl	0.2172	0.2141	0.2202	b
34 °C	Pl	0.2129	0.2098	0.2160	bc
28 °C	Vs	0.1897	0.1868	0.1926	d
30 °C	Vs	0.2145	0.2116	0.2174	b
32 °C	Vs	0.2320	0.2291	0.2349	a
34 °C	Vs	0.2156	0.2127	0.2185	b

Pl = *Plantago*; Vs = *Veronica*

Table S5: Linear model for individual fat content (related to Figure 2B in the main text). **A**) Minimum adequate model (in bold) was obtained using the *step()* function, starting from the full model. **B**) Anova table for the minimum adequate model. **C**) Model-estimated marginal means, as well as upper and lower confidence limits, for all experimental treatments. Significant differences between experimental groups (Tukey's HSD, α = 0.05), averaged over the families, were obtained using the package *emmeans* and are indicated by different letters.

**A**		AIC
**M_{full}**	**Fat content ~ F * T * HP**	**184.1**
M_2	Fat content ~ F + T + HP + F:T + F:HP + T:HP	236.5

F = family; T = temperature; HP = host plant.

**B**	Df	Sum Sq	Mean Sq	F value	P value	% exp
Family	13	175.3395	13.4877	12.1996	< 0.0001	11.4766
Temperature	3	95.9078	31.9693	28.9162	< 0.0001	6.2775
Plant	1	33.0864	33.0864	29.9267	< 0.0001	2.1656
Family:Temperature	39	204.0132	5.2311	4.7315	< 0.0001	13.3534
Family:Plant	13	102.8951	7.9150	7.1591	< 0.0001	6.7349
Temperature:Plant	3	16.7780	5.5927	5.0586	0.0018	1.0982
Family:Temperature:Plant	39	134.7115	3.4541	3.1243	< 0.0001	8.8174
Residuals	692	765.0626	1.1056			50.0763

**C**	Temperature	Plant	Mean	LCL	UCL	Group
	28 °C	Pl	7.0044	6.8002	7.2086	a
	30 °C	Pl	7.1823	6.9734	7.3912	a
	32 °C	Pl	7.6754	7.4670	7.8838	b
	34 °C	Pl	7.8885	7.6758	8.1012	b
	28 °C	Vs	6.7918	6.5859	6.9977	a
	30 °C	Vs	6.7932	6.5857	7.0007	a
	32 °C	Vs	6.7812	6.5715	6.9909	a
	34 °C	Vs	7.7098	7.5061	7.9136	b

Pl = *Plantago*; Vs = *Veronica*

Table S6: Linear model for individual development time (related to Figure S2A in the supplementary materials). **A**) Minimum adequate model (in bold) was obtained using the step() function, starting from the full model. **B**) Anova table for the minimum adequate model. **C**) Model-estimated marginal means, as well as upper and lower confidence limits, for all experimental treatments. Significant differences between experimental groups (Tukey's HSD, α = 0.05), averaged over the families, were obtained using the package emmeans and are indicated by different letters.

**A**		AIC
**M_{full}**	**Development time ~ F * T * HP**	**1417.6**
M_2	Development time ~ F + T + HP + F:T + F:HP + T:HP	1477.7

F = family; T = temperature; HP = host plant.

**B**	Df	Sum Sq	Mean Sq	F value	P value	% exp
Family	13	734.6413	56.5109	24.338672	< 0.0001	13.2852
Temperature	3	185.8150	61.9383	26.676229	< 0.0001	3.3603
Plant	1	13.3771	13.3771	5.7614	0.0165	0.2419
Family:Temperature	39	361.1477	9.2602	3.988275	< 0.0001	6.5309
Family:Plant	13	566.0942	43.5457	18.754705	< 0.0001	10.2372
Temperature:Plant	3	7.2857	2.4286	1.045963	0.3712	0.1318
Family:Temperature:Plant	39	310.9925	7.9742	3.434394	< 0.0001	5.6239
Residuals	1443	3350.4369	2.3219			60.5889

**C** Temperature	Plant	Mean	LCL	UCL	Letter
28 °C	Pl	15.749	15.5310	15.9677	bcd
30 °C	Pl	15.297	15.0809	15.5122	c
32 °C	Pl	15.326	15.1041	15.5487	c
34 °C	Pl	16.098	15.8717	16.3241	ad
28 °C	Vs	15.875	15.6611	16.0883	db
30 °C	Vs	15.492	15.2816	15.7014	bc
32 °C	Vs	15.337	15.1255	15.5479	c
34 °C	Vs	16.393	16.1801	16.6061	a

Pl = *Plantago*; Vs = *Veronica*

Table S7: Linear model for individual diapause mass (related to Figure S2B in the supplementary materials). **A**) Minimum adequate model (in bold) was obtained using the *step()* function, starting from the full model. **B**) Anova table for the minimum adequate model. **C**) Model-estimated marginal means, as well as upper and lower confidence limits, for all experimental treatments. Significant differences between experimental groups (Tukey's HSD, α = 0.05), averaged over the families, were obtained using the package *emmeans* and are indicated by different letters.

**A**		AIC
**M_{full}**	**Diapause mass ~ F * T * HP**	**2891.9**
M_2	Diapause mass ~ F + T + HP + F:T + F:HP + T:HP	2938.8

F = family; T = temperature; HP = host plant.

**B**	Df	Sum Sq	Mean Sq	F value	P value	% exp
Family	13	3564.6910	274.2070	45.7602	< 0.0001	19.4455
Temperature	3	3797.6067	1265.8689	211.2507	< 0.0001	20.7160
Plant	1	169.6805	169.6805	28.3166	< 0.0001	0.9256
Family:Temperature	39	758.5425	19.4498	3.2458	< 0.0001	4.1379
Family:Plant	13	608.4506	46.8039	7.8107	< 0.0001	3.3191
Temperature:Plant	3	63.2488	21.0829	3.5184	0.0146	0.3450
Family:Temperature:Plant	39	722.6794	18.5302	3.0924	< 0.0001	3.9422
Residuals	1443	8646.8305	5.9923			47.1687

**C** Temperature	Plant	Mean	LCL	UCL	Letter
28 °C	Pl	6.8730	6.5222	7.2238	e
30 °C	Pl	9.0712	8.7248	9.4176	d
32 °C	Pl	10.0496	9.6925	10.4067	bc
34 °C	Pl	10.6042	10.2408	10.9677	bf
28 °C	Vs	7.1585	6.8154	7.5016	e
30 °C	Vs	9.5695	9.2323	9.9067	cd
32 °C	Vs	11.3837	11.0444	11.7230	a
34 °C	Vs	11.2182	10.8760	11.5603	af

Pl = *Plantago*; Vs = *Veronica*
